# The Relationships among Plasma Fetuin-B, Thyroid Autoimmunity, and Fertilization Rate In Vitro Fertilization and Embryo Transfer

**DOI:** 10.1155/2022/9961253

**Published:** 2022-03-16

**Authors:** Rui Zhang, Feifei Cheng, Wei Cheng, Xin Wang, Binghan Zhang, Mingyuan Tian, Ke Li, Dongfang Liu

**Affiliations:** ^1^Department of Endocrinology and Metabolism, The Second Affiliated Hospital of Chongqing Medical University, Chongqing, China; ^2^Department of Endocrinology and Metabolism, The Chongqing People's Hospital, Chongqing, China

## Abstract

**Objective:**

The objective of the study is to investigate the relationships between fetuin-B, thyroid autoimmunity (TAI), and pregnancy outcomes in women undergoing in vitro fertilization and embryo transfer (IVF-ET). *Design, Patients, and Measurements*. In this prospective study, 180 women who were preparing for pregnancy with IVF-ET were included. There were 120 women with TAI positive and 60 negative controls matched with age and BMI.

**Results:**

The 180 women had mean ± SD age of 31.4 ± 4.0 years, with a mean ± SD BMI of 21.0 ± 1.6 kg/m^2^. There was a significant difference in the level of fetuin-B in women with TAI positive compared with TAI negative group (65.2 ± 18.5 vs. 76.4 ± 25.1, *P*=0.001). Fetuin-B had a negative relationship with thyroid antibodies even after adjusting for other variables (OR (95%CI) = 0.98 (0.96–0.99), *P*=0.002). Compared with women with TAI negative, those with TAI positive had a significantly higher risk of low fertilization (20.0% vs. 6.7%; *P*=0.035). And we found no difference in terms of pregnancy, abortion, implantation, and live birth rate between the two groups. Logistic regression analysis showed that both fetuin-B and TAI were the independent factors to lead the low fertilization of IVF-ET (OR (95%CI) = 0.96 (0.94–0.99) and 4.084 (1.39–15.30), *P*=0.004 and 0.019, respectively).

**Conclusion:**

Fetuin-B was significantly associated with TAI and low fertilization rate in women undergoing IVF-ET. Decreased fetuin-B in women with TAI may be the underlying reason for the lower IVF-ET success rate.

## 1. Introduction

Fetuins are members of the cystatin proteases inhibitor superfamily, including fetuin-A and fetuin-B [1]. Previous studies reported that fetuin-A was associated with anti-inflammatory, inhibiting tumour growth, angiogenesis, regulating body calcification, and insulin resistance [2]. Fetuin-B possesses a 22% homology with fetuin A. Fetuin-B deficiency in female mice rendered completely infertile because of premature zona pellucida hardening [3]. Dietzel and colleagues [4] found that serum fetuin-B was associated with fertilization rate in women undergoing in vitro fertilization and embryo transfer (IVF-ET), and the level of fetuin-B might be a useful predictor for successful fertilization in IVF-ET.

Thyroid autoimmunity (TAI) is characterized by the positive thyroid peroxidase antibody (TPOAb) and/or thyroglobulin antibody (TgAb) with or without the thyroid dysfunction. TAI is very common in women of reproductive age, accounting for 8–14% worldwide [5]. Besides, infertile women had a higher risk of TAI compared with healthy fertile women [6, 7]. Previous studies found that there was a decreased trend of fertilization rate, implantation rate, and clinical pregnancy rate but increased abortion rate in women with TAI in IVF-ET, compared with those without [8–10].

In our recent study, we collected the follicular fluid samples from euthyroid patients with positive or negative thyroid antibodies undergoing IVF-ET and applied proteomics to identify the differentially expressed proteins between the two groups. Our results showed that the level of fetuin-B in follicular was significantly different between women with TAI positive and controls [11]. Fetuin-B levels in serum and follicular fluid are tightly associated [4]. However, the relationship between fetuin-B and TAI, as well as with pregnancy outcomes, in women undergoing IVF-ET has not been examined thoroughly. In this study, we aimed to explore (1) the relationship between plasma fetuin-B and TAI; (2) the predictive role of fetuin-B on pregnancy outcomes in women with and without TAI; and (3) the possible interaction between TAI and fetuin-B on pregnancy outcomes of IVF-ET.

## 2. Material and Methods

### 2.1. Subjects

In this cohort study, we consecutively recruited women who visited the reproductive endocrine clinics at the Second Affiliated Hospital of Chongqing Medical University (Chongqing, China) between January 2019 and July 2020. All subjects were screened for thyroid autoimmunity. A total of 120 euthyroid women with thyroid autoimmunity positive were included in this study, as well as 60 healthy controls matched with age and body mass index (BMI). The eligible subjects had primary infertility because of male factor, tubal factor, or both and were planning to receive IVF-ET.

Exclusion criteria included current smokers, the history of thyroid surgery, known other female causes related to reproductive difficulty, the use of antithyroid drug or thyroid hormone, and subjects who were previously diagnosed with overt thyroid diseases such as Graves' disease, hypothyroid, hypertension, diabetes mellitus, Cushing's syndrome, malignant tumour, polycystic ovary syndrome (PCOS), ovarian insufficiency, or other autoimmune diseases such as antiphospholipid syndrome. The study was approved by the Ethics Committee of Second Affiliated Hospital of Chongqing Medical University, and informed written consent was obtained from all subjects.

### 2.2. Clinical, Anthropometrical, and Biochemical Parameters

A standard questionnaire was used to document personal records including age, medical, and drug history. Height (cm) and weight (kg) were obtained at baseline, and BMI was calculated as BMI (kg/m^2^) = weight (kg)/height (m)^2^. After an 8-12-hour overnight fast, venous blood was collected for measuring thyroid function, TPOAb, and TgAb (Elecsys Autoanalyzer, Roche Diagnostics). Plasma fetuin-B level was tested by the enzyme-linked immunosorbent assay kits (No. SEB860Hu, Cloud-Clone Corp., Houston, TX, USA). The sensitivity of the assay was 59 pg/ml and the linear range of the standard was 125–8000 pg/ml. The intra-assay variation was <10%, and the interassay variation was <12%. These measurements were performed by well-trained research experts, and the analytical performance of these assays was within the specifications of the analyzers. Blood samples for measuring sex hormones were collected during the early follicular phase of menstrual bleeding (3rd to 5th days) in the morning before the controlled ovarian hyperstimulation (COH) treatment. Chemiluminescence method was used to determine estradiol (E2), luteinizing hormone (LH), follicle-stimulating hormone (FSH), prolactin (PRL), antimüllerian hormone (AMH), progesterone (Prog) (LIAISON XL analyzer, DiaSorin, Italy), and testosterone (TEST) (Abbott Company kit, Abbott i2000 analyzer, USA).

### 2.3. In Vitro Fertilization and Embryo Transfer Protocols

The following scheme was used to promote ovulation in the subjects in this study: a gonadotropin-releasing hormone agonist (GnRH-a, 1 mg/d) was injected in the middle luteal period of the patient's previous menstrual cycle for 14–18 days, which could compete with GnRH in the pituitary gland, preventing GnRH release, reducing the production of associated hormones in the ovary, and resulting in pituitary downregulation. At the same time, recombinant follicle-stimulating hormone (rFSH, 150–300IU/d) was given for ovarian stimulation according to individual ovarian response estimated by serum estradiol concentrations and follicular growth by ultrasound. When there were at least 2 leading follicles ≥18 mm in diameter, women were injected with the 10000IU human chorionic gonadotropin (hCG) to induce ovulation. Oocytes were transvaginally retrieved under ultrasound guidance 36h after approximate administration. IVF or intracytoplasmic sperm injection (ICSI) was carried out for fertilization depending on semen parameters. After 3 days from ovum retrieval, embryo transfer was performed, and progesterone was administered for the luteal support.

### 2.4. Definition of Pregnancy Outcomes

Fertilization condition was defined as the presence of pronuclei in the retrieved oocytes under the microscope, 16–18 h after conventional insemination. The normal fertilization rate was defined as the percentage of two pronuclei per the total number of retrieved oocytes. Low fertilization rate (LFR) was defined as a fertilization rate less than 20% of normal [12]. The embryos implantation rate was calculated from the number of implanted embryos divided by the total number of transferred embryos. Clinical pregnancy was identified by the fetal heartbeats detected at transvaginal ultrasound examination performed 5–6 weeks after ovum pickup. Abortion was defined as the loss of pregnancy. Live birth was defined as the delivery of a fetus with signs of life after 24 completed weeks of gestational age. We also collected the number of oocytes retrieved, available embryos, and embryos transferred.

### 2.5. Statistical Analysis

Data analysis was performed with the Statistical Package for the Social Sciences, version 25 (SPSS Inc., Chicago, USA) and *R* version 3.6.1 (http://www.r-project.org). Data are expressed as mean ± SD, median (Q1, Q3), or proportion (%), as appropriate. Variables with skewness less than ±1 were considered as following a normal distribution, and covariates were natural logarithmic transformed if skewed. Comparison for numeric variables between groups was performed using Student's *t*-test or Mann–Whitney *U* test. Chi-square or Fisher's exact tests were used as appropriate for categorical variables. Correlations between the level of fetuin-B and other variables were quantified using Pearson and partial correlations. Logistic regression analysis was conducted to analyse the relationship between fetuin-B and the clinical outcomes of IVF-ET. *P* values < 0.05 (two-tailed) were considered statistically signiﬁcant.

## 3. Results

### 3.1. Clinical Profiles of Study Subjects

The 180 women had mean ± SD age of 31.4 ± 4.0 years, with a mean ± SD BMI of 21.0 ± 1.6 kg/m^2^, a median infertility duration of 3.0 [Q1-Q3: 2.0–5.0] years, and 66.7% (*N* = 120) being TAI positive (Table 1). There were 109 (60.6%) women with TPOAb positive and 100 (55.6%) with TGAb positive. A total of 89 (49.4%) women had both increased levels of TPOAb and TGAb. No significant differences concerning age, BMI, FT3, FT4, AMH, sex hormone, causes of infertility, and duration of infertility were observed between women with TAI positive versus negative. Compared with TAI negative, the level of thyroid-stimulating hormone (TSH) was higher in TAI positive (2.7 1 ± 1.00 vs. 2.37 ± 0.76, *P*=0.020). There were only 15 women underwent ICSI procedure, while others received IVF procedure. There was no significant difference in low fertilization rate, pregnancy, abortion, implantation, and live birth rate between women with IVF or ICSI (14.6% vs. 26.7%, *P*=0.258; 38.2% vs. 40.0%, *P*=1.000; 16.4 % vs. 20.0%, *P*=0.719; 62.4% vs. 53.3%, *P*=0.582; 15.2% vs. 13.3%, *P*=1.000, respectively).

Compared with women with TAI negative, those with TAI positive had a significantly higher risk of low fertilization (20.0% vs. 6.7%; *P*=0.035). We did not observe any significant difference in the percentage of implantation, clinical pregnancy, and live birth between the two groups. In addition, subjects in TAI positive group had a decreasing trend of available embryos than those in the TAI negative group; however, this difference did not reach the statistical significance (5.5 ± 2.7 vs. 6.3 ± 2.8, *P*=0.071) (Table 1).

### 3.2. Relationship between Fetuin-B and Thyroid Antibodies Status

**Figure 1:**
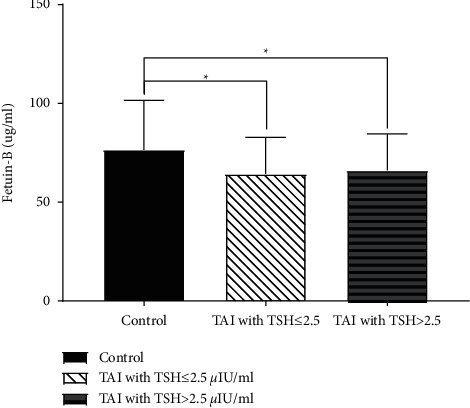
The comparison of fetuin-B between patients with TAI positive and controls. There were 56 (46.7%) patients with TSH >2.5 *μ*IU/ml among women with TAI positive (*N* = 120). We did not observe a significant difference in the level of fetuin-B between TAI group with different thyroid functions (64.20 ± 18.66 in TSH >2.5 *μ*IU/ml group vs. 66.02 ± 18.54 in TSH ≤2.5*μ*IU/ml group, *P*=0.884). Women with TAI negative (60 controls, 76.43 ± 25.08) had a significant higher level of fetuin-B than those with TAI positive regardless of the different levels of TSH (*P*=0.006 and 0.017, separately). ^*∗*^*P* < 0.05.

The relationship between fetuin-B and baseline characteristics was presented in Supplementary Table 1. Fetuin-B was inversely associated with TPOAB and TGAB in this study (*r* = -0.207 and -0.218; *P*=0.005 and 0.003, respectively). Fetuin-B in women with TAI positive was significantly lower than the control group (65.2 ± 18.6 vs. 76.4 ± 25.1, *P*=0.001). The decreasing trend of fetuin-B level was independent of the thyroid function (64.20 ± 18.66 in TSH >2.5 *μ*IU/ml group and 66.02 ± 18.54 in TSH ≤2.5*μ*IU/ml group vs. 76.43 ± 25.08 in controls, *P*=0.006 and 0.017, respectively) (Figure 1). The relationship between fetuin-B and TAI status is presented in Table 2. Our results suggested that fetuin-B had a negative relationship with thyroid antibodies even after adjusting for other variables (OR = 0.98, *P*=0.002).

### 3.3. Pregnancy Outcomes

Logistic regression analysis was conducted to evaluate the relationship between fetuin-B, thyroid antibodies status, and their interaction effect on pregnancy outcomes. The result showed that fetuin-B was an independent factor leading to the low fertilization (OR (95%CI) = 0.96 (0.94–0.99), *P*=0.004). TAI was also significantly associated with the low fertilization (OR (95%CI) = 4.08 (1.39–15.30), *P*=0.019) (Table 3).

**Figure 2:**
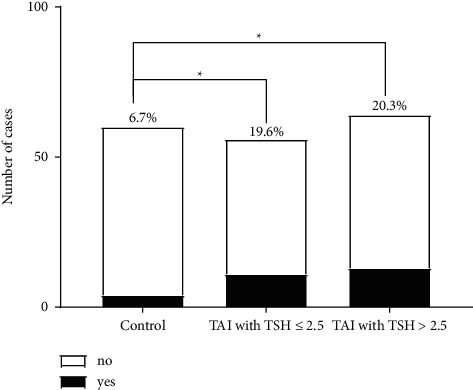
The comparison of fertilization rate between patients with TAI positive and controls.

Due to the difference of TSH between the control and the TAI positive group, we further made a comparison of IVF results among groups including control, TAI with TSH≤ 2.5*μ*IU/ml, and TAI with TSH>2.5*μ*IU/ml. There were no significant differences in terms of pregnancy, abortion, implantation, and live birth. As expected, the low fertilization rate was 6.7% in the control group, as compared with 19.6% and 20.3% in the TAI group with TSH≤ 2.5*μ*IU/ml and TSH>2.5*μ*IU/ml (*P*=0.037 and 0.027, respectively) (Figure 2).

Besides, we tested the interactions between fetuin-B and TAI status on the pregnancy outcomes. The *P* values for interaction analysis of fetuin-B with TAI status were only significant for the outcome of live births (*P*=0.013, Table 3).

## 4. Discussion

The association between TAI and adverse pregnancy outcomes has been a significant topic, and while a few studies have established a superficial relationship, they have not been able to pinpoint the underlying reason [13–15]. In this study, we may be able to provide a new perspective on this problem. Women with TAI positive had a lower level of plasma fetuin-B, which was independent of the thyroid function. We found that the lower fertilization rate was observed in TAI-positive women, and both the fetuin-B and TAI were the independent predictive factors for pregnancy outcome in women undergoing IVF-ET.

Up to date, there have been several prevailing explanations regarding the TAI and adverse pregnancy outcomes. Firstly, some researchers have reported that the presence of thyroid autoantibodies has a close link to thyroid hormones deficiency, which is obvious when thyroid function cannot compensate for the increased stress during the pregnancy [16, 17]. Besides, the COH applied to assisted reproductive technology, will induce the elevated concentration of serum estrogen, lead to a rise in thyroxine-binding globulin (TBG), and finally induce the decrease of free thyroxine (FT4) and increase of TSH [13, 18]. In our study, we observed a higher level of TSH in women with TAI, but no difference was observed in the TAI group with TSH> 2.5*μ*IU/mL or TSH <2.5*μ*IU/mL in the matter of pregnancy outcome. In a previous study, Weghofer et al. [14] demonstrated that the embryo quality might be impaired when TSH presents as high normal (TSH >2.5*μ*IU/mL). Fumarola et al. [15] also concluded that the mild elevation of TSH (TSH >2.5*μ*IU/mL) badly affects the clinical pregnancy rate in women undergoing IVF-ET. However, Busnelli et al. [19] reported that there was no difference in the outcome of pregnancy, implantation, and delivery between women with clinical or subclinical hypothyroid treated with levothyroxine and the euthyroid. Reh et al. [20] suggested that TSH thresholds of ≥2.5*μ*IU/mL and ≥4.0*μ*IU/mL brought no difference in pregnancy outcomes after IVF-ET. Therefore, further studies were needed to investigate the correlation among thyroid antibodies, thyroid function, and the pregnant outcomes of IVF-ET.

We found that the level of fetuin-B was lower in women with TAI than those without and was inversely related to the level of thyroid antibodies. And the results showed that the level of TSH did not affect the fetuin-B level in the TAI positive group. In addition, the level of fetuin-B was significantly associated with pregnancy outcomes in IVF-ET, which was independent of traditional risk factors. Finally, we observed the interaction between fetuin-B and TAI. Based on our previous research, we identified the downregulated fetuin-B in follicular with TAI positive [21]. Therefore, we believed that the lower level of fetuin-B was associated with the low fertilization rate in the TAI group.

Fetuin-B, as an inhibitor of ovastacin, was reported to contribute to the achieved fertilization in IVF-ET by preventing zona pellucida hardening (ZPH) [22]. The zona pellucida (ZP) composed of four zona proteins (ZP1-ZP4) is an extracellular glycoprotein matrix surrounding growing oocytes. It works as a species-specific sperm barrier, controlling the binding of single sperm, the induction of the acrosomal reaction, and the promotion of sperm-egg fusion [23, 24]. ZP hardening was caused by the function of ovastacin proteases on proteolytic of ZP glycoproteins, particularly the cleavage of ZP2 to ZP2f [25–27]. The premature ZP hardening is bad for fertilization because it will shorten the time window of oocyte fertilization [28]. In vitro fertilization cycles, we can see the occurrence of premature ZPH in the process of in vitro maturation (IVM) which has been taken as a reason for the lower fertilization rate [29]. And some researchers indicated that if premature ZPH can be prevented, any form of assisted reproductive treatment will benefit from it [30, 31].

In an animal study by Floehr et al. [32], they treated 12 mice (6–13 weeks old) with repetitive fetuin-B antisense oligonucleotides (ASO) to downregulate hepatic synthesis and decrease the level of serum fetuin-B and then mated them with the normal fertile male. Their results showed that only one of twelve got pregnant, but all of these mice got pregnant again after withdrawing the treatment of ASO to normalize the level of serum fetuin-B. In addition, they conducted the IVF protocol in five mice to evaluate the influence of different levels of fetuin-B on the success of IVF, concluding that the serum fetuin-B below 10ug/ml will influence the fertilizable state of oocyte which may give a new clue on the contraceptive medication for female [32].

From our results, we suspect that the thyroid antibodies may decrease the concentration of fetuin-B in plasma and may also influence the level of fetuin-B in follicular fluid, finally affecting the success of fertilization. However, examining this hypothesis may be beyond the scope of the current analysis.

The process of fertilization regulated by different signalling molecules is complicated. Previous studies reported that the thyroid tissue may possess similar antigens with zona pellucida, so the circulation thyroid antibodies can take the zona pellucida as the targeted tissue to bind on, which directly influences the oocyte-sperm interaction [33]. On the other hand, it was suggested that the inflammation would increase the failure rate of IVF-ET, and the pro- and anti-inflammatory cytokines may badly affect the production of good-quality embryos and hinder embryo implantation [34, 35]. And some studies showed that patients with thyroid antibodies had increased inflammation markers indicating that the thyroid autoimmunity may be related to the chronic inflammation [36, 37].

We found that the low fertilization rate was higher in the TAI group than control, and both fetuin-B and thyroid antibodies are independent factors in the outcome of LFR. This result may be partly explained by the direct effect of thyroid antibodies on fertilization and the indirect effect through decreasing the level of fetuin-B in plasma. Therefore, the significant correlation between thyroid antibodies and fetuin-B may provide a new perspective on the negative effect of thyroid antibodies towards the pregnancy outcome of IVF-ET.

In regard to the strengths and limitations of the current study, this is an extension of the current follicular fluid research, further exploring the relationship between fetuin-B and thyroid antibodies in the blood, providing new clues for the interpretation of the effects of thyroid antibodies on assisted reproduction outcomes. However, we were unable to study the effect of TSH on pregnancy outcomes since the small sample size. TAI and TSH screening were measured only at one point before IVF-ET, which limited our ability to explore the impact of their dynamic change on the occurrence of adverse pregnancy outcomes.

## 5. Conclusion

Plasma fetuin-B, a strong factor affecting the fertilization rate, was closely related to thyroid antibodies and showed a decreasing trend in women with TAI positive. Thyroid autoimmunity played a critical role in low fertilization rate possibly by direct actions and influencing the level of fetuin-B in plasma. Decreased fetuin-B in women with TAI may be the underlying reason for the lower IVF-ET success rate. As such, further investigations are required to identify if thyroid antibodies combined with fetuin-B can be a predictive marker for the success of fertilization in women with IVF-ET.

## Figures and Tables

**Table 1:** Baseline characteristics of subjects according to the status of thyroid antibodies.

Variables	TAI positive	TAI negative	*P* value
	(n = 120)	(n = 60)	
Age (years)	31.43 ± 3.79	31.45 ± 4.32	0.979
BMI (kg/m^2^)	20.97 ± 1.63	21.07 ± 1.53	0.692
Infertility duration (years)	3.00 [2.00, 5.00]	3.00 [2.00, 5.00]	0.704^*∗*^
Infertility causes (%)			
Female factor	88 (73.3%)	43 (71.7%)	
Male factor	18 (15.0%)	11 (18.35%)	
Combined factor	14 (11.7%)	6 (10.0%)	
TSH (*μ*IU/ml)	2.71 ± 1.00	2.37 ± 0.76	0.020
Free T3(pmol/l)	4.60 ± 0.58	4.69 ± 0.58	0.292
Free T4(pmol/l)	16.92 ± 2.70	16.82 ± 2.04	0.783
TPOAb (IU/ml)	280.57 ± 197.98	17.68 ± 6.96	<0.001
TgAb (IU/ml)	391.00 [189.55, 618.35]	20.30 [17.50, 24.10]	<0.001^∗^
LH (IU/L)	4.44 ± 1.30	4.65 ± 1.48	0.352
FSH (IU/L)	7.18 ± 1.60	6.90 ± 1.49	0.259
LH/FSH	0.64 ± 0.20	0.68 ± 0.19	0.148
Estradiol (pmol/L)	37.87 ± 10.09	39.85 ± 10.68	0.225
Prolactin (mIU/L)	17.68 ± 5.36	17.40 ± 4.93	0.731
Testosterone (nmol/L)	21.29 ± 8.08	21.67 ± 8.83	0.770
AMH (ng/ml)	3.19 ± 1.02	3.41 ± 1.19	0.198
Oocytes retrieved (N)	12.03 ± 3.70	11.93 ± 3.73	0.876
Available embryos(N)	5.49 ± 2.73	6.28 ± 2.81	0.071
Embryos transferred (N)	1.82 ± 0.53	1.82 ± 0.43	0.916
Low fertilization (%)	24 (20.0%)	4 (6.7%)	0.035^#^
Implantation (%)	71 (59.2%)	40 (66.7%)	0.416^#^
Pregnancy (%)	42 (35.0%)	27 (45.0%)	0.255^#^
Abortion (%)	23 (19.2%)	7 (11.7%)	0.289^#^
Live birth (%)	19 (15.8%)	8 (13.3%)	0.825^#^
Fetuin-B (ug/ml)	65.17 ± 18.54	76.43 ± 25.08	0.001

All data are expressed as mean ± SD, median [Q1-Q3], or proportion in %; ^*∗*^Mann–Whitney *U* test was used in infertility duration and TgAb.^#^Chi-square or Fisher's exact tests were used as appropriate for categorical variables. AMH, anti-Müllerian hormone; BMI, body mass index; Free T3, free triiodothyronine; Free T4, free thyroxine; FSH, follicle-stimulating hormone; LH, luteinizing hormone; TgAb, thyroglobulin antibody; TPOAb, thyroid peroxidase antibody; TSH, thyroid-stimulating hormone.

**Table 2:** The relationship between fetuin-B and thyroid antibodies status.

Variables	Model 1		Model 2		Model 3		Model 4	
	OR (95%CI)	*P* value	OR (95%CI)	*P* value	OR (95%CI)	*P* value	OR (95%CI)	*P* value
Intercept	11.33 (3.79–36.79)	<0.001	17.86 (0.16–2037.68)	0.227	4.96 (0.04–639.42)	0.514	37.94 (0.15–11263.07)	0.203
Fetuin-B (ug/ml)	0.98 (0.96–0.99)	0.001	0.98 (0.96–0.99)	0.002	0.98 (0.96–0.99)	0.001	0.98 (0.96–0.99)	0.002
Age (years)			1.00 (0.92–1.09)	0.997	1.00 (0.92–1.09)	0.970	0.99 (0.90–1.08)	0.832
BMI (kg/m^2^)			0.98 (0.80–1.20)	0.832	0.99 (0.80–1.22)	0.897	0.97 (0.79–1.21)	0.811
TSH (*μ*IU/ml)					1.53 (1.08–2.23)	0.021	1.53 (1.07–2.23)	0.024
LH/FSH							0.25 (0.05–1.31)	0.100
AMH (ng/ml)							0.87 (0.63–1.20)	0.384

AMH, anti-Müllerian Hormone; BMI, body mass index; FSH, follicle-stimulating hormone; LH, luteinizing hormone; OR, odds ratio; TSH, thyroid-stimulating hormone. Model 1: without adjustment. Model 2: adjusted for age and BMI. Model 3: Model 2 + adjusted for TSH. Model 4: Model 3 + adjusted for AMH and the ratio of LH and FSH.

**Table 3:** The relationship between fetuin-B, thyroid antibodies status, and their interaction effect on pregnancy outcomes.

Outcomes	Fetuin-B (ug/ml)		TAI status		*P* value for interaction
	OR (95%CI)	*P* value	OR (95%CI)	*P* value	
Low fertilization	0.96 (0.94–0.99)	0.004	4.08 (1.39–15.30)	0.019	0.616
Implantation	1.00 (0.99–1.02)	0.927	0.73 (0.37–1.42)	0.359	0.157
Pregnancy	1.01 (0.99–1.02)	0.300	0.66 (0.35–1.26)	0.203	0.691
Abortion	1.00 (0.99–1.02)	0.684	1.91 (0.79–5.16)	0.171	0.836
Live birth	0.99 (0.96–1.01)	0.209	1.13 (0.47–2.93)	0.790	0.013

OR, odds ratio; TAI, thyroid autoimmunity. The fully adjusted model was adjusted for age, BMI, infertility duration, and AMH. Fetuin-b, TAI status, and their interaction were included in the model separately.

## Data Availability

The datasets generated during and/or analysed during the current study are available from the corresponding author on reasonable request.

## Supplementary Materials

Supplementary Materials Supplementary Table 1: the relationship between fetuin-B and baseline characteristics.
