# Supplementary material for: The Relationships among Plasma Fetuin-B, Thyroid Autoimmunity, and Fertilization Rate In Vitro Fertilization and Embryo Transfer
Source: Int J Endocrinol. 2022 Mar 16;2022:9961253. doi: 10.1155/2022/9961253 (PMC8942698; doi:10.1155/2022/9961253)
Supplement: Supplementary Materials — Supplementary Table 1: the relationship between fetuin-B and baseline characteristics. [file 9961253.f1.docx]

Table S1. The relationship between Fetuin-B and baseline characteristics

| Variables |  | **Fetuin-B (ug/ml)** | | |
| --- | --- | --- | --- | --- |
|  |  |  | adjusting for age | adjusting for age and BMI |
| Age (years) | r | -0.004 |  |  |
|  | P value | 0.961 |  |  |
| BMI (Kg/m^2^) | r | 0.047 | 0.049 |  |
|  | P value | 0.528 | 0.516 |  |
| Ln (Infertility duration) | r | 0.041 | 0.053 | 0.050 |
|  | P value | 0.584 | 0.478 | 0.506 |
| TSH（μIU/ml） | r | -0.012 | -0.012 | -0.011 |
|  | P value | 0.873 | 0.872 | 0.883 |
| Free T3 (pmol/l) | r | 0.076 | 0.078 | 0.077 |
|  | P value | 0.310 | 0.301 | 0.305 |
| Free T4 (pmol/l) | r | 0.108 | 0.108 | 0.109 |
|  | P value | 0.149 | 0.149 | 0.146 |
| TPOAb (IU/ml) | r | -0.207 | -0.207 | -0.214 |
|  | P value | **0.005** | **0.005** | **0.004** |
| Ln (TgAb) | r | -0.218 | -0.219 | -0.219 |
|  | P value | **0.003** | **0.003** | **0.003** |
| LH (IU/L) | r | 0.023 | 0.023 | 0.027 |
|  | P value | 0.757 | 0.757 | 0.725 |
| FSH (IU/L) | r | 0.089 | 0.089 | 0.092 |
|  | P value | 0.237 | 0.238 | 0.220 |
| LH/FSH | r | -0.049 | -0.049 | -0.047 |
|  | P value | 0.515 | 0.517 | 0.530 |
| Testosterone (nmol/L) | r | 0.101 | 0.102 | 0.103 |
|  | P value | 0.176 | 0.175 | 0.173 |
| Estradiol (pmol/L) | r | 0.099 | 0.099 | 0.099 |
|  | P value | 0.188 | 0.189 | 0.188 |
| prolactin (mIU/L) | r | 0.000 | 0.000 | 0.000 |
|  | P value | 0.997 | 0.999 | 0.999 |
| AMH (ng/ml) | r | 0.123 | 0.129 | 0.135 |
|  | P value | 0.100 | 0.085 | 0.072 |

Natural logarithmic transformations were used in Infertility duration and TgAb, due to the skewed distribution.

Abbreviation: AMH, anti-Müllerian Hormone; BMI body mass index; Free T3, free triiodothyronine; Free T4, Free thyroxine; FSH, follicle-stimulating hormone; LH, luteinizing hormone; r, Pearson correlation coefficient; TgAb, thyroglobulin antibody ; TPOAb, thyroid peroxidase antibody; TSH, thyroid-stimulating hormone.
